# Developing Normative Integration among Professionals in an Intersectoral Collaboration: A Multi-Method Investigation of an Integrated Intervention for People on Sick Leave Due to Common Mental Disorders

**DOI:** 10.5334/ijic.4694

**Published:** 2019-11-04

**Authors:** Rie Mandrup Poulsen, Kathrine Hoffmann Pii, Ute Bültmann, Mathias Meijer, Lene Falgaard Eplov, Karen Albertsen, Ulla Christensen

**Affiliations:** 1 Copenhagen Research Center for Mental Health – CORE, Rehabilitation, Recovery and Shared Care, Mental Health Center Copenhagen, Mental health services in the Capital Region of Denmark, Hellerup, DK; 2 University College Copenhagen, Faculty of Health, Department of Nursing and Nutrition, København N, DK; 3 University of Groningen, University Medical Center Groningen, Department of Health Sciences, Community and Occupational Medicine, Groningen, NL; 4 Work and health, TeamWorkingLife Aps, Valby, DK; 5 Department of Public Health, Section of Social Medicine, University of Copenhagen, København K, DK

**Keywords:** integrated care, normative integration, shared goals, vocational rehabilitation, mental health care, intersectoral collaboration

## Abstract

**Introduction:**

Intersectoral integration is recommended in vocational rehabilitation, though difficult to implement. We describe barriers to and strategies for the development of normative integration in an intersectoral, team-based vocational rehabilitation intervention.

**Method:**

Attitudes and behaviours regarding the development of shared culture, norms, and goals in the collaboration between health care professionals and employment consultants were investigated through 30 semi-structured interviews, participant observation of 12 intersectoral meetings, and document analysis of 12 joint plans.

**Results:**

Organisational factors and unsettled power balance between professionals constituted barriers to the development of a shared culture. These issues were resolved by establishing smaller work teams, and through health care professionals’ gradual acceptance of employment consultants’ control in their capacity as administrators of legislation. Some barriers to shared norms were resolved explicitly, whereas implicit diverging norms were continuously negotiated. The development of shared goals was supported by clarifying the fit between individual, professional, and organisational goals, though the alignment of goals required a paradigmatic change of mindset among the health care professionals.

**Conclusion:**

This study shows how normative integration among health care professionals and employment consultants is feasible in co-located intersectoral teams, with positive implications for the delivery of coherent support.

## Introduction

Facilitating integration between the health care and social sectors is important but challenging [1,2,3,4]. This challenge is prominent in the area of vocational rehabilitation for people with mental disorders where multidisciplinary support is recommended to help individuals recover their mental health and re-enter the labour market [5,6,7]. Service providers and stakeholders involved in vocational recovery come from health care, social services, occupational health services, social or private insurance companies, as well as workplaces and trade unions [5,8]. They often have different plans, success criteria and expectations for the individual on sick leave [8,9,10], which can cause increased stress for the person on sick leave [11]. Lack of coordination between stakeholders is problematic for mentally ill persons [12] who frequently experience loss of control, decreased planning capabilities, and poor predictability regarding their recovery [13].

Horizontal integration in vocational rehabilitation has been initiated during the last 15–20 years, e.g. through case coordination, inter-agency meetings, multidisciplinary teams, and co-location [14]. Research indicates that these integration activities can be hindered by a lack of effective communication, clear service goals, trust, commitment, and integrated leadership [5,14]. Differences in values among professionals have been identified as a key barrier to integration in vocational rehabilitation for people with mental disorders [15,16]. Integration through the alignment of goals, values, culture, and norms has been termed *normative Integration* [17]. Though shared goals and values are suggested to be important determinants of behaviour and decision-making, and thus integral to integration [18,19], the intangible concept of *normative integration* has proven difficult to investigate [17,20,21]. This study will address a gap in research on integrated care [21] by examining the development of normative integration between sectors that lack naturally overlapping goals and values.

### The Danish IBBIS project

In 2015, The Danish Agency for Labour Market and Recruitment and the Ministry of Employment initiated the ‘Integreret Behandlings- og BeskæftigelsesIndsats til Sygedagpengemodtagere med stress, angst og depression’ (IBBIS) project in collaboration with The Mental Health Services of the Capital Region of Denmark and four *Jobcenters*. The aim was to develop and test a new model for integrated mental health care and vocational rehabilitation for persons on sick leave due to depression, anxiety, and stress disorders. The IBBIS intervention provided a novel model for integration between the institutions (designated as the host organisations) of the health care and social sectors. Persons on sick leave (designated as IBBIS participants) received integrated support from care managers (from the public mental health care services) and employment consultants (from the *Jobcenters*).

### Aim

This study investigates barriers to the development of normative integration among care managers and employment consultants, and how these barriers were handled. By presenting the employed coping strategies, we aim to inspire researchers and practitioners who engage in designing and implementing normative integration.

### Empirical setting

The IBBIS intervention was financed primarily by the Ministry of Employment and, to a lesser degree, by the four involved municipal *Jobcenters*. No expenses were involved for the Mental Health Services of the Capital Region of Denmark. Public *Jobcenters* are described as municipal-level one-stop-shops within the social sector and concurrently administer social policies and labour market policies concerning all citizens on sick leave for minimum four weeks [22,23].

**Figure 1:**
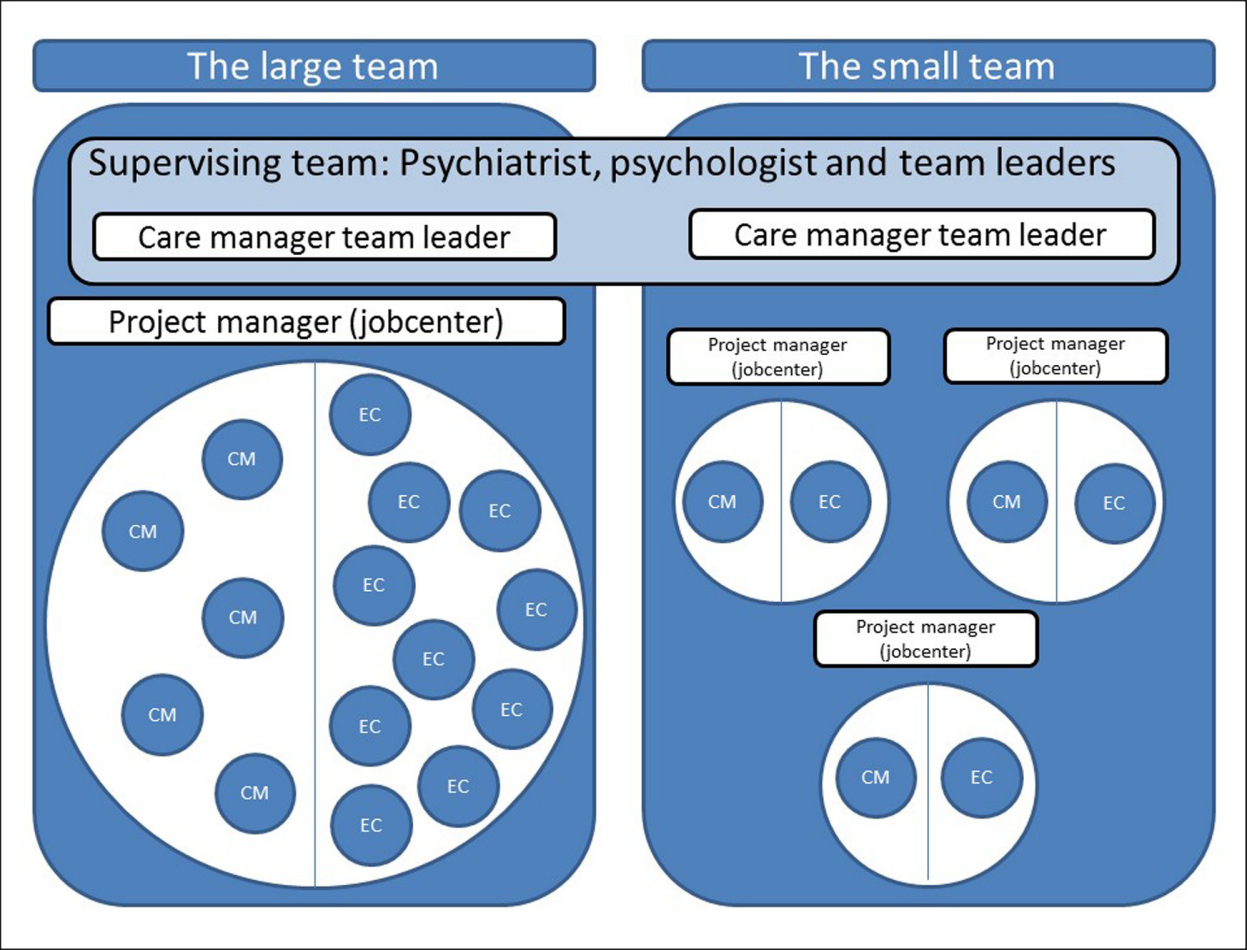
Initial organisation of care managers (CM), employment consultants (EC), and supervisors in two IBBIS teams.

The intervention was delivered in two multicentre, randomized controlled trials with return to any work without receipt of sick leave benefit as the primary outcome, from May 2016 to November 2018 [24,25]. Care managers provided therapy or stress coaching. Mental health care centers have no direct responsibility to support return to work, and most care managers had no previous experience with work-oriented intervention or collaboration with the Jobcenters. Employment consultants provided vocational rehabilitation support based on problem-solving methods [24,25,26] based on the Dutch ‘SHARP- at work’ intervention [27,28], gradual return to work (which is the common method for return to work for employed persons in Denmark), and managed the sick leave case in accordance with Danish sick leave legislation. Administration of the public sickness benefit insurance case involves continuous monitoring of workability to decide eligibility for financial support [29,30]. Thus, employment consultants both support and control persons on sick leave [31]. All facilities were provided by the municipalities which the *Jobcenters* belonged to. The IBBIS intervention was delivered by two teams, henceforth designated as *The large team* and *The small team*. *The large team* consisted of 10 part-time employment consultants (EC), five care managers (CM), and one team leader/CM. *The small team* consisted of three full-time employment consultants, three care managers, and one team leader/CM (see Figure 1). All professionals were supervised by the team leader, a psychiatrist, and a psychologist, who were employed in the mental health care services, and supported by administrative project managers from the *Jobcenters*.

Care managers and employment consultants in *The small team* worked together in dyads, while care managers in *The large team* initially worked with all (up to eight part-time) employment consultants in the teams. Employment consultants in *The large team* divided their work hours evenly between the regular *Jobcenter* and the IBBIS intervention. Care managers and employment consultants will subsequently be referred to as professionals.

### Program theory of integration in the IBBIS intervention

The program theory of integration in the IBBIS intervention was based on Jody Hoffer Gittell’s middle range theory of *relational coordination* [32]. Relational coordination is developed through an iterative process where *shared goals, shared knowledge*, and *mutual respect* between functional groups lead to communication that is *often, timely, accurate, understandable, and problem-solving*, and vice versa [33,34]. The creation of shared goals among professionals and participants was described as particularly important in the integrated IBBIS intervention and should be established through inter-disciplinary assessment of the participant in the context of a roundtable meeting [24,25,26]. During the roundtable meeting, the IBBIS participant, the care manager, and the employment consultant should describe the goals and plans for the participant after each professional had made a mono-disciplinary assessment. The roundtable meeting resulted in a written document, *The joint plan*, which described the mono-disciplinary and shared goals and plans [26]. See [24,25,26] for an elaborate description of integration activities. The intervention manual was revised 11 months into the intervention delivery [26]. Selected alterations will be addressed in the results and discussion sections.

## Theory and method

### Theoretical framework

Data collection and analysis were initially driven by concepts from the program theory, with an emphasis on shared goals. As recommended in the process evaluation literature [35,36], secondary theory was used to elicit contextual factors that influenced the implementation. To capture the influences of the host-organisations that were unexpected or unexplained by the intervention program theory, we have drawn on the concept of normative integration [17]. The analytical framework consists of three important aspects of normative integration: *shared culture, shared norms*, and *shared goals*. Normative integration is hypothesised to be important at the individual, professional, organisational, and system levels [37]. The creation of a shared culture across sectors with coherent norms and goals for practice is hypothesised to facilitate coherent services [37] and prevent conflicting approaches towards the end-user [38]. We have applied the concept of *shared norms* to cover *collective attitude* and *transcending domain perceptions* [37].

### Method

This study is part of a process evaluation of the IBBIS intervention, which builds on recommendations from the British Medical Research Council [39]. The roundtable meeting was chosen as the primary empirical setting because ideally it forms an arena for creating shared goals and plans for the IBBIS participant [26]. Practices and attitudes relating to the collaboration were investigated 12–22 months into the intervention delivery through 1) observations of 12 IBBIS participants’ first roundtable meeting; 2) 24 interviews with the participating care managers and employment consultants conducted after the meetings; 3) document analysis of 12 *joint plan* documents; and 4) three interviews with supervisors. To improve the long-term developmental perspective, we conducted follow-up interviews with three supervisors 27 months into the intervention. See appendix 1 for an overview of interviews, observations, and informant characteristics and appendix 2 for additional methodological descriptions. These qualitative methods provided a broad description of the professionals’ verbal and written collaboration, the ideals for this collaboration, and the contextual factors that affected it. The 12 IBBIS participants and associated professionals were purposively selected to cover both IBBIS teams and all three dyads in *The small team*. The purpose of comparing practices from the two teams was to enhance the understanding of the possible influence of contextual factors, such as the different cultural contexts of the four involved *Jobcenters*. Some professionals were observed and interviewed twice to describe their collaboration with other professionals. All data material was collected by RMP.

#### Observation of roundtable meetings

Participant observation (n = 12) of roundtable meetings with 12 IBBIS participants, care managers, and employment consultants was conducted to study the professionals’ practices during these meetings. Furthermore, the observations provided information about significant behaviours and expressions during the roundtable, which were addressed during the individual interviews to gain insight into the professionals’ interpretation of their practices.

#### Individual interviews with professionals and supervisors

We conducted 30 interviews: 24 interviews with the professionals who participated in the 12 roundtable meetings 12–22 months into the intervention delivery, (see appendix 1). Supervisors and team leaders, all employed in the mental health services, were interviewed twice: first after 14–16 months into intervention delivery in order to add their perspectives on the social dynamics of the two teams (n = 3) and second after 27 months into intervention delivery (n = 3) in order to enhance the long-term developmental perspective and the description of solutions and coping strategies (follow-up interviews are indicated in quotes in the results section). The 30 interviews lasted on average 55 (27–93) minutes. All interviews were recorded and transcribed verbatim, and RMP coded the interview material using Nvivo 11 software.

#### Document analysis of *Joint Plans*

The *joint plans* provided a summary of the 12 observed roundtable meetings and included the participants’ written plan for the mental health care and vocational rehabilitation interventions, as well as the shared goals and overall plan for the integrated intervention [26]. The *Joint plan* documents were collected before the individual interviews so that concrete goals could be addressed in the interviews and were used to analyse the types of goals that were noted following the roundtable meetings.

#### Analytical approach

The semi-structured interviews were analysed using systematic text condensation (STC), which is a four-step thematic analysis. Central to STC is the decontextualisation by coding of text across interviews and informants, the condensation of codes into meaning units, and the thematisation of these units [40]. In this study, the coding and thematisation were done abductively [41]. Sub-codes were empirically grounded, and some sub-codes were organised under global codes that were theoretically loaded (e.g. the sub-code *return to work as primary goal* was coded under the global code *shared goals*). However, the whole data material was coded to remain sensitive to emerging themes, e.g. about contextual organisational factors. In accordance with the STC approach, data were analysed continuously throughout data collection, preliminary codes were created, and the interview- and observation guide were adjusted halfway through data collection [40]. Field notes and *joint plan* documents were interpreted with a focus on the expression of the display of goals and norms, and possible discrepancies between care managers and employment consultants. To enhance sensitivity, UC has reviewed codes for selected interview material [42]. KHP and RMP have discussed themes and findings to enhance reflexivity and challenge preconceptions and interpretations [43].

## Results

Interviews, observations, and documents showed that shared culture, norms and goals were negotiated and developed between the professionals, their host organisations, and IBBIS participants. We describe the barriers in this development and the coping strategies that were applied.

### Shared work culture

A shared work culture between care managers and employment consultants developed quickly among some professionals, while others experienced the collaboration as somewhat problematic.

#### Unsettled balance and competition between professionals

For some professionals, the unsettled power balance between care managers and employment consultants appeared as a barrier to the development of a shared work culture. Care managers’ and employment consultants’ positioning towards each other was not explicitly articulated during the initial interviews, but it came across in observations and particularly in follow-up interviews with supervisors. A supervisor described this positioning as a competition over the right to “own the process”, i.e. to define and control the IBBIS participant’s process. The competition was also described in terms of the professional’s bond with the participant:

“Sometimes they [employment consultants] needed to make themselves interesting, if they had been too passive with a case. […] I think it sometimes felt like a competition. You know, who had the best relationship with the participant. […] In the beginning, we sort of had to sound each other out. I think it has become less of an issue”. (Care manager, The large team)

This positioning between care managers and employment consultants took place in the context of discussions about the appropriate balance between *health* and *employment*. Problems of balance were described concretely in terms of how much the different functional groups talked during meetings, and, more abstractly, the extent to which the groups were recognised by one another and by supervisors. Some professionals (both care managers and employment consultants) initially felt that this balance tipped in favour of the other functional group.

Competition and positioning subsided during the intervention period. The competition about owning the participants’ process redounded to the employment consultants’ advantage because of their power over the IBBIS participants’ benefits. This was demonstrated during a roundtable meeting when an employment consultant had determined a sick leave benefit case in clear disagreement with the care managers, who had no formal mandate to challenge that decision. A supervisor described how the care managers ultimately ended up in a less powerful position:

“I actually think we believed that the health care professionals would have more of a say in all this. We just thought, well, we go and do therapy, and hand them over [to the employment consultants] whenever we think they are well enough. […] Employment consultants have the power to close the money box, and that’s why they have the power.” (Supervisor, follow-up interview)

Sick leave legislation turned out to be an important factor in the participant’s process of recovering and returning to work. Care managers’ professional judgment was not backed by a legislative mandate, and this affected the power balance. An employment consultant described the role of legislation:

“We meet in this context, and the context is the sick leave benefit legislation. That’s the framework for this intervention.” (Employment consultant, The large team)

However, one supervisor suggested that the wish to ‘own the case’ subsided when divisions of responsibility became clearer, and management confirmed areas where each professional would accept to share control:

“We could agree that this is something that’s decided by care managers, and this is decided by *Jobcenter* professionals, and we cannot object to that, and thirdly that there are some things that we need to agree on, and then we would try to agree. Then it’s easier to focus on the task.” (Supervisor, follow-up interview)

This furthermore shows that sharing control in some areas might depend on clearly defining certain areas where each professional has the mandate and competencies to make decisions on their own.

#### Number of intersectoral relationships, team size, and part-time workers

Furthermore, a large team size and a perceived high number of intersectoral relationships (up to eight employment consultants for each care manager) was perceived as a barrier to integration. A care manager said:

“There was constant confusion. You have to relate to 24 participants in the first place. You spend so much time establishing all these relations. You get confused by all these different people whom you have to relate to in your head.” (Care manager, The large team)

A supervisor argued that the dyadic intersectoral relationships in *The small team* were more independent of the culture in the host organisations and thus provided better opportunities for developing a new shared culture. The relationships in *The small team*, where only two professionals worked together, were described as “you and I”, whereas they were characterised as “us and them” in *The large team* because working cultures were established within each sector. Though engaging in only one intersectoral relationship presented obvious benefits, some professionals and supervisors found that two-person teams also posed a certain risk in terms of personal chemistry:

“This *arranged marriage* is a bit interesting. […] There are pros and cons, because we have an opportunity to fine-tune the collaboration between two people, and that’s all good, but do you like each other personally? It’s very vulnerable.” (Care manager, The small team)

Employment consultants’ part-time positions in *The large team* were considered problematic by supervisors and professionals because they posed practical problems, hindered availability, and ultimately delayed the development of a group identity and shared culture. An employment consultant found the change in workplaces demanding:

“I’m the kind of person who needs to settle in each time I change environment. You know, I just need to remember, who was that participant? What were the routines around here? I just need to adjust. […] I just need to remind myself, this is where the coffee is, these are my colleagues here […]. It takes a lot of energy.” (Employment consultant, The large team)

These challenges were addressed by splitting the large team into smaller intersectoral groups. According to professionals and supervisors, this improved the collaboration. However, the part-time positions were upheld throughout the intervention delivery to maintain employment consultants’ focus on legislation and regular *Jobcenter* practice. The establishment of smaller teams allowed professionals to gain shared experiences with each professional from the other sector. Nonetheless, some care managers felt that the routine built from these experiences could not easily be transferred to collaborations with other professionals as approaches differed too much between individual professionals, e.g. because employment consultants interpreted the sick leave legislation differently.

### Shared norms

Care managers and employment consultants carried norms that were dominant in their host organisations. Conflicting organisational norms constituted a barrier to intersectoral collaboration, which professionals and supervisors dealt with in different ways.

Some diverging norms were addressed directly. The different way of referring to IBBIS participants as *patients* in the mental health care services and as *citizens* in the *Jobcenters* was considered problematic in the integrated intervention, and the term *citizen* was quickly chosen by management, and used in the revised manual. Followingly, care managers consistently used the term citizens during interviews. Other norms were explicitly discussed by professionals and management, e.g. the norms for intersectoral supervision of professionals. Care managers and employment consultants favoured the norms from mental health care where reflective supervision prevails, rather than the more case-based supervision commonly used in *Jobcenters*. This issue was settled when management supported the professionals’ wishes, and only reflective supervision was provided.

Other more subtle discrepancies in norms were continuously negotiated, like diverging norms for how comfortable the IBBIS participant should feel in the intervention, and how this was best accomplished by professionals. Care managers described how most IBBIS participants feared interaction with professionals from the *Jobcenter*, and how the mentioning of demands and rules could create an uncomfortable atmosphere. Some care managers felt that they needed to compensate for this through their own communication, or through encouraging employment consultants to communicate legislative demands less often, or less directly. However, some employment consultants found the meetings sufficiently comfortable and argued that their focus on sick leave legislation – though uncomfortable to discuss – was necessary to avoid unrealistic expectations.

Therefore, sick leave legislation sometimes limited the extent to which meetings with the IBBIS participant could be perceived as comfortable. Some employment consultants addressed the enduring differences in norms by dissociating themselves somewhat from the norms of their host organisation. A care manager described how an employment consultant defined her practice in opposition to regular *Jobcenter* practice:

“This [kind of sick leave case management] would never have occurred, as she tells me, down there [in the regular *jobcenter*]. They would not have gone that far with the case”. (Care manager, The small team)

Some professionals described employment consultants’ ‘flexibility’ in interpreting the sick leave legislation as a positive contribution to the intersectoral working relationship since it showed that they held similar views regarding the interests of the IBBIS participant. However, other employment consultants did not display any flexibility in this regard and found that the successful collaboration with care managers and supervisors depended on the latter respecting the superior role of legislation:

“Everybody learned something in this project. They [mental health care professionals] pitied the ones [participants] who were transferred to the assessment program [lower benefit level]. But there is nothing about pity in the legislation. It’s very black and white. But they [care managers and supervisors] respect it now.” (Employment consultant, The small team)

The two quotes show that the continuous negotiation produced different practices, and that each professional created different norms for each intersectoral relationship. Furthermore, the locally negotiated norms created a sense of uncertainty in the collaboration, particularly during roundtable meetings:

“Though I have done a lot of these meetings, I still don’t feel like I have much experience with it. It [the roundtable meeting] differs so much depending on which employment consultant I work with.” (Care manager, The large team)

### Shared goals

In line with this, professional’s views on service goals were not initially overlapping. Interviews, observations, and documents showed that several different goals were at stake in the delivery of the IBBIS intervention. The roundtable meeting formed a potential arena for the identification of a suitable fit between the respective goals of the care managers, employment consultants, and IBBIS participants. However, these goals also had to match those of the two host organisations, and the intervention goal described in the manual. A supervisor described how the professionals had to comply with several sets of ‘rules’ and find a compromise:

“They [employment consultants] belong to an organisation where legislation represents a very obvious guiding principle. It’s sort a fundamental condition for their work. That’s something they cannot just disclaim. Then they are introduced to some new rules in the IBBIS project. They say okay and make an effort to live up to this. And that’s fine. But sometimes they forget them, and sometimes the new rules do not fit the old ones. The same goes for care managers.” (Supervisor)

Observations showed that care managers’ goals with the therapy were often made verbally explicit through phrases like ‘we aim to improve her ability to say no’, whereas the vocational rehabilitation goal of returning to work or entering new employment was tacitly accepted during the meeting. The roundtable meeting was designed to promote the development of a person-centered goal with the intervention. However, observations showed that the written *shared goals* for IBBIS participants were often formulated after the meeting by one of the professionals who wrote or copy pasted a sentence about returning to employment, which reflected the IBBIS intervention goal. Formulating a shared goal for the IBBIS participant was initially conceived as a bureaucratic technicality. It was often done through email correspondence between the professionals and did not guarantee a shared conception of intervention goals.

The IBBIS intervention goal – to support the participants’ return to employment – was adopted differently by care managers and employment consultants. Most employment consultants accepted the predefined IBBIS intervention goal, which they found meaningful and in agreement with their regular goals at the *Jobcenter*. An employment consultant even described the two goals in unity:

“That will always be the goal. Returning to work. Closing the benefit case.” (Employment consultant, The large team)

In contrast, only a few care managers had previously perceived the improved workability as an essential goal with their therapy, but they had accepted the intervention goal as an inevitable premise for helping the participant. However, some care managers initially expressed doubts about the therapeutic implications of this goal. Care managers always formulated a separate treatment goal, which was perceived as more meaningful by some care managers. The link between the treatment goal and the work-related goal was not always clear:

“I can’t really see how it fits. Because my overall goal is for her to recover from her depression and be able to make better decisions in the future. She is all wound up about some things, rigid and perfectionist, and I’m thinking, for me the goal is to get her out of her perfectionism, and I don’t really know what part her work plays in that.” (Care manager, The large team)

This indicates that the written goal of the IBBIS intervention described in the *Joint plan* – most often ‘return to work’ at a specific date – was not initially accepted as a shared goal. This barrier to normative integration resolved after the relationship between the care managers’ therapeutic goal and the vocational goal was negotiated. Vocational goals were given superior status, e.g. when supervisors’ and employment consultants described vocational goals as ‘framework’ goals. This ‘goal hierarchy’ was communicated through supervision and the revised intervention manual in which therapeutic goals were defined as supportive of vocational goals. In the first version of the manual, the care manager goal was “to cure the mental condition and, if that is not possible, to secure the best possible recovery process for the participant with the overall goal of enabling the participant to live a satisfying and independent life”. In the second version, the care manager goal had become “to support the final goal of the IBBIS intervention, i.e. that the participant returns to work”. Supervisors described care managers’ willingness to shift their focus from symptom-related goals to work-related goals in their therapeutic work as a *paradigmatic* shift:

“We now see things in a more similar way. At least that’s the case now, after one and a half years. It has not always been like that. This is something that needs continuous attention. We keep talking about ‘work as treatment’ and those dilemmas. […] But this is all new to the care managers. Before [in regular mental health care] it was about keeping the patient calm and providing care. This is a paradigmatic shift, to place therapy in a ‘return to work’ framework.” (Supervisor, follow-up interview)

The supervisors’ phrase ‘work as treatment’ expressed the rationale that work would ultimately provide social meaning, daily structure, and economic security for participants. This justified the vocational goal as an advantage, not only for society, but for the individual participant. The individual benefits of work participation was important for care manager’s acceptance of the superior status of the vocational goals and coming to peace with their supportive role in the IBBIS intervention.

## Discussion

Our study suggests that normative integration developed among IBBIS professionals through a series of interrelated strategies and solutions throughout the 31 months of intervention delivery. We found that the unsettled professional power balance, the high number of intersectoral working relationships, and part-time positions constituted barriers to the development of a shared work culture among care managers and employment consultants. Factors which supported a shared work culture included the settling of informal power positions, individual professionals’ willingness to share and relinquish control, the splitting-up of a large team, and the generation of shared experiences. Whereas some differences in professional and organisational norms were addressed explicitly and resolved directly by management, other more subtle discrepancies continued to be negotiated, with negative implications for the collaborative effort. Several diverging goals co-existed. The establishment of the project goal – to support return to work – as the superior goal provided coherence and the care managers’ willingness to submit to this paradigm proved crucial.

Our study suggests that the negotiation of norms and goals among professionals was affected by norms and goals on macro level (e.g. political goals to restrict access to sick leave benefits), the meso-level (e.g. goals to comply with sick leave legislation), and the micro-level (like the participants’ goal to receive financial support), as suggested by Valentijn [37]. New norms were established in a combination of top-down and bottom-up processes [44], and some remained negotiated at the professional level (See Table 1).

**Table 1:** Barriers to normative integration among professionals, types of coping processes, and their implications (care managers: CM; employment consultants: ECs).

	Barriers	Coping strategies	Types of strategies	Possible implications

**Shared culture**	Positioning and unsettled power balance between CMs and ECs	Informal hierarchy in which ECs have more power and control due to their legislative mandate	Macro-level influence through legislation	Overly unbalanced relationships might jeopardise engagement
		ECs and CMs accepted to share control in some aspects	Meso-level negotiation	Informal power balance up for negotiation
	High number of intersectoral relationships and part-time positions	Development of smaller intersectoral teams	Organisational change	Vulnerability (personal chemistry, staff turnover, holiday) in very small teams
		Working relationships established through shared experiences with each professional	Person-based collaboration	Time-consuming process

**Shared norms**	Diverging terminology for the person on sick leave	Management decision in favour of *Jobcenter* terminology	Top-down (confirmed by revised manual)	Acceptance by IBBIS professionals
	Norms for supervision	Management decision to comply with mental health care approach to supervision, prompted by demands from CMs and ECs	Bottom-up	Acceptance and satisfaction among professionals
	Norms for professional approach during roundtable meetings	Negotiated with each professional	Meso-level negotiation	Perceived unpredictability between professionals

**Shared goals**	Diverging professional, organisational, and project goals	Clear hierarchy between professional goals (documented in the revised manual)	Top-down (confirmed by the revised manual)	CMs are expected to be rather flexible Overly unbalanced relationship might jeopardise engagement
		Paradigmatic shift in mindset among health care professionals facilitated by supervision		

### Alignment of goals to support persons on sick leave

The importance of shared goals among professionals, organisations, and systems that engage in integrated services has been highlighted in integration research [37,45]which distinguishes six integration dimensions (clinical, professional, organisational, system, functional and normative integration. However, research has shown that stakeholders in the return to work field are characterised by having diverging goals for the person on sick leave [46,47]. Typically, employers and social insurance organisations have financial incentives to shorten sick leave periods, whereas the health care sector in most countries has little motivation to improve vocational rehabilitation and often puts mental recovery goals above work-related goals [6,47,48]. Our study supports the assumption that return to work interventions serve several interests simultaneously, and we suggest the concept of *goal-pluralism* to describe this phenomenon. We argue that the implementation of integration was delayed by a lack of managerial focus on this *goal-pluralism*. Franche and colleagues have argued that different paradigmatic views on the return to work process cannot realistically be harmonised [49]. However, integration was eventually facilitated by the change in intervention goals manifested in the revised manual. Though care managers and employment consultants did not end up with identical goals, the alignment with intervention goals improved. Some professionals compensated for the differences in goals and norms by dissociating themselves from their own original goals and those of their organisation. Our study shows that care managers’ increasing acceptance of a ‘work-as-treatment paradigm’ contributed to the alignment of goals, which has been emphasised in vocational rehabilitation research [5]. We suggest that the care managers’ supportive role was negotiated and settled during the delivery of the intervention, and that this established role clarity, which is proven crucial for inter-organizational collaboration [50]. This study attests to the importance of role clarity for inter-organisational collaboration. We furthermore suggest that clarity on a possible hierarchy of goals, explicit descriptions of the professional’s latitude for using service user goals directionally in the intervention, and training in appropriate goal-setting could be beneficial for normative integration.

### Balance between systems

This study suggests that goals, norms, and cultures emanating from the mental health care centres and the *Jobcenters* reflect different rationales, which were negotiated and partly merged in the IBBIS team throughout the delivery of the intervention delivery. Craig and colleagues recommend that contextual factors like financial and political interests should be taken into account in process evaluations [36]. This was only done indirectly in the present study. This study addresses the neglected topic of the balance of power between the involved professionals and their host organisations. As described earlier, the level of financial involvement differed between the social service sector (the Ministry of Employment and *Jobcenters* provided funding for both care manager and employment consultant services) and the health care sector (the Mental Health Care Services of Copenhagen provided no funding). We suggest that the power balance between the professionals was affected by the uneven financial and managerial interests of their host organisations. The monetary flow from the social and employment sectors to the health care sector could potentially create a costumer-supplier relationship between mental health care services and the *Jobcenters*, and we suggest this might counteract normative integration.

### Limitations

The developmental focus in this study evolved throughout data collection, since establishing working relationships proved to be a long-term process [50]. We initially prioritised high information power [51] in the comparison between the two teams and between IBBIS participants with different conditions to describe organisational differences and diverging practices relating to the establishment of goals during roundtable meetings. However, follow-up interviews with care managers and employment consultants might have illuminated the process to an even larger extent. The longitudinal perspective was, however, covered by the three follow-up interviews with supervisors, which described the developmental aspect of the integration process. Furthermore, patients’ perspectives on normative integration are not addressed directly in this study. This was due partly to the need to focus on selected stakeholders, and partly to the program theory of the intervention in which relational coordination between professionals was pivotal. However, patient perspectives on the integrated IBBIS intervention will be described in a separate study.

Integration of services to support persons on sick leave due to common mental disorders will take very different organisational forms in different countries, and this study shows its implications in a particular political, legal, and organisational context [36]. In the Danish general coverage system, public mental health care centres and *Jobcenters* are highly relevant to integration. Organisations similar to the *Jobcenters* are only found in other comprehensive social security systems [52]. Nonetheless, our results might be relevant to other integrated care interventions where stakeholder incentives and values are not naturally overlapping [18].

### Implications for practice and research

This study indicates that future similar interventions should place great managerial importance on the (possible) fit between stakeholders’ goals in newly established integration interventions. When intervention developers and implementers state their goals, a prioritisation of goals might minimise conflicts between professionals. Furthermore, this has the potential to elicit imbalances that might jeopardise engagement from under-favoured organisations.

The time it takes to establish shared goals and norms and a shared culture between individual professionals should not be underestimated, and the pros and cons of operating with a high number of intersectoral relationships should be considered. In line with recommendations on team structure in supported employment [53], we propose that intersectoral teams could meet the balance between nourishing intersectoral relationships and avoiding problems with small teams (e.g. related to staff illness or maintaining high intra-sectoral professional skills) by recommending a team-size between 4 and 10.

This study was not designed to investigate the goals and norms of stakeholders at neither the organisational (e.g. top hospital management) nor the system levels (e.g. policy makers). However, there appears to be increasing political interest in integrated services in the social service and health care sectors in Denmark and other western countries [47,48,54]. We suggest that future research investigate the interests and goals of stakeholders at the organizational and system levels in order to explore the possibilities for normative integration at the top-level [37].

## Conclusion

This study suggests that normative integration among professionals from the health care and social sectors is feasible in co-located intersectoral teams, with positive implications for the delivery of coherent support for people on sick leave due to common mental disorders. We found that the initially unsettled power balance between care managers and employment consultants, and the perceived high number of intersectoral relationships, acted as barriers for a shared culture. The development of a shared culture across sectors was supported by the division of one team into smaller units, by health care professionals’ acceptance of employment consultants’ legally mandated authority, and by the clear division of professionals’ responsibilities and areas of control. Some barriers to the establishment of shared norms were resolved explicitly, whereas implicit diverging norms were continuously negotiated. The development of shared goals was facilitated by explicating how individual, professional, organisational, and system goals align and by health care professionals’ willingness to bridge possible differences through a paradigmatic shift of mindset. The structural influence of sick leave legislation was initially under-recognised, but ultimately it strongly affected the negotiation of a shared culture, norms and goals.

## Additional Files

The additional files for this article can be found as follows:

10.5334/ijic.4694.s1 Appendix 1. Overview of observations, and interviews and informant characteristics.

10.5334/ijic.4694.s2 Appendix 2. Supplementary information regarding observations and interviews.
