# Supplementary material for: Developing Normative Integration among Professionals in an Intersectoral Collaboration: A Multi-Method Investigation of an Integrated Intervention for People on Sick Leave Due to Common Mental Disorders
Source: Int J Integr Care. 2019 Nov 4;19(4):4. doi: 10.5334/ijic.4694 (PMC6838772; doi:10.5334/ijic.4694)
Supplement: Appendix 1. — Overview of observations, and interviews and informant characteristics. [file ijic-19-4-4694-s1.pdf]

Appendix 1: Overview of involved informants, observations and interview

IBBIS participant	Participant gender	Participant sick leave cause	Observation of roundtable meeting	Care manager interview date	Position in IBBIS	Employment consultant interview date	Position in IBBIS	Team
**P 1**	Male	Stress	20-04-2017	CM 1: 05-05-2017	Full time	EC 1: 28-04-2017	Full time	The small team
**P 2**	Male	Anxiety	02-05-2017	CM 2: 04-05-2017	Full time	EC 2: 09-05-2017	50 %	The large team
**P 3**	Woman	Depression	08-05-2017	CM 3: 10-05-2017	Full time	EC 3: 08-05-2017	50 %	The large team
**P 4**	Woman	Depression	15-05-2017	CM 2: 23-05-2017	Full time	EC 4: 23-05-2017	50 %	The large team
**P 5**	Woman	Depression	16-05-2017	CM 3: 31-05-2017	Full time	EC 3: 22-05-2017	50 %	The large team
**P 6**	Woman	Stress	24-05-2017	CM 4: 01-06-2017	Full time	EC 5: 24-05-2017	50 %	The large team
**P 7**	Male	Stress	24-10-2017	CM 5: 24-10-2017	CM/team leader	EC 6: 01-11-2017	Full time	The small team
**P 8**	Male	Stress	31-10-2017	CM 6: 08-11-2017	Full time	EC 7: 08-11-2017	Full time	The small team
**P 9**	Woman	Stress	16-11-2017	CM 7: 16-11-2017	Full time	EC 6: 30-11-2017	Full time	The small team
**P 10**	Woman	Stress	22-11-2017	CM 8: 24-11-2017	Full time	EC 8: 29-11-2017	50 %	The large team
**P 11**	Male	Stress	30-11-2017	CM 5: 05-12-2017	Full time	EC 7: 05-12-2017	Full time	The small team
**P 12**	Woman	Stress	11-01-2018	CM1: 11-01-2018	Full time	EC 1: 17-01-2018	Full time	The small team

Table 1: Observations and interviews evolving around 12 IBBIs participants, and informant characteristics

Table 2: Initial and follow-up interviews with the supervisor team

Supervisor interview	Initial interview	Follow-up interview
**Supervisor/team leader 1:**	04.09.2017	13.07.2018
**Supervisor/team leader 2:**	01.07.2017	02.07.2018
**Supervisor/team leader 3**	30.08.2017	
**Supervisor/team leader 3**		11.07.2018
