# Supplementary material for: Developing Normative Integration among Professionals in an Intersectoral Collaboration: A Multi-Method Investigation of an Integrated Intervention for People on Sick Leave Due to Common Mental Disorders
Source: Int J Integr Care. 2019 Nov 4;19(4):4. doi: 10.5334/ijic.4694 (PMC6838772; doi:10.5334/ijic.4694)
Supplement: Appendix 2. — Supplementary information regarding observations and interviews. [file ijic-19-4-4694-s2.pdf]

Appendix 2: Supplementary method description

Observations

With a focus on the dialogue and the interactions during the roundtable meeting, we wished to investigate the professional's roles in the facilitation of the meeting and their roles towards the IBBIS participant. To elucidate the professional's explicit or implicit goals with the IBBIS intervention, the observations were furthermore centered around the discussion on plans and goals and the process of translating this into the *Joint Plan* document.

Interviews

The interview guide focused on the professionals' expectations and experiences with the roundtable meeting, and their perception of goals with the services, general working relationship, and coordination of the IBBIS intervention. Contextual factors such as organizational differences were addressed through questions about the professional's attitudes and experiences with the two host organizations.
